# THRIVE study protocol: a randomized controlled trial evaluating a web-based app and tailored messages to improve adherence to adjuvant endocrine therapy among women with breast cancer

**DOI:** 10.1186/s12913-019-4588-x

**Published:** 2019-12-19

**Authors:** Andrew J. Paladino, Janeane N. Anderson, Rebecca A. Krukowski, Teresa Waters, Mehmet Kocak, Carolyn Graff, Ryan Blue, Tameka N. Jones, Joanne Buzaglo, Gregory Vidal, Lee Schwartzberg, Ilana Graetz

**Affiliations:** 1 Department of Health Policy and Management, Emory University, Rollins School of Public Health, 1518 Clifton Road, Atlanta, GA USA; 2 The West Cancer Center & Research Institute, Memphis, TN USA; 3 College of Nursing, The University of Tennessee Health Science Center, 920 Madison Avenue, Memphis, TN USA; 4 Department of Preventive Medicine, The University of Tennessee Health Science Center, College of Medicine, 66 N Pauline St, Memphis, TN USA; 5 Department of Health Management and Policy, The University of Kentucky, College of Public Health, Lexington, KY USA; 6 Department of Patient Reported Outcomes, Vector Oncology, Memphis, TN USA

**Keywords:** Randomized controlled trial, Adjuvant endocrine therapy, Breast cancer, Medication adherence, Mhealth, Patient-reported outcomes, Quality of life

## Abstract

**Background:**

Long-term use of adjuvant endocrine therapy (AET) among women with early-stage, hormone receptor-positive breast cancer significantly reduces the risk of hospitalizations, cancer recurrence, and mortality. AET is associated with adverse symptoms that often result in poor adherence. A web-enabled app offers a novel way to communicate and manage symptoms for women on AET. In a region with significant racial disparities in breast cancer outcomes, our study tests the impact of a web-enabled app that collects and transmits patient-reported symptoms to healthcare teams to facilitate timely and responsive symptom management on medication adherence.

**Methods:**

In this randomized controlled trial, we randomize 300 patients initiating AET to one of three arms: 1) an “App” group (*n* = 100) that receives weekly reminders to use the THRIVE study app; 2) an “App+Feedback” group (*n* = 100) that receives weekly reminders and tailored feedback based on their use of the app; or 3) a “Usual Care” group (*n* = 100) that receives usual care only. Participants are stratified by race: 50% White and 50% Black. The duration of the intervention is six months following enrollment, and outcomes are assessed at 12-months. The primary outcome is adherence, which is captured using an electronic monitoring pillbox. Secondary outcomes include symptom burden, quality of life, self-efficacy for managing symptoms, and healthcare costs. We also evaluate the impact of the intervention on racial disparities in adherence. Data are derived from three sources: electronic health record data to capture treatment changes, healthcare utilization, and health outcomes; self-report survey data related to adherence, symptom burden, and quality of life; and an electronic medication monitoring device that captures adherence.

**Discussion:**

A successful web-enabled intervention could be disseminated across systems, conditions, and populations. By evaluating the impact of this intervention on a comprehensive set of measures, including AET adherence, patient outcomes, and costs, our study will provide valuable and actionable results for providers, policy makers, and insurers who strive to achieve the “Triple Aim” – reduce costs while improving health outcomes and the patient care experience.

**Trial registration:**

NCT03592771. Prospectively registered on July 19, 2018.

## Background

More than 80% of U.S. women with breast cancer have hormone receptor-positive (HR+) tumors [1]. They are commonly prescribed adjuvant endocrine therapy (AET) after surgery, chemotherapy and/or radiation [2, 3]. Long-term use of AET significantly reduces the risk of hospitalizations, cancer recurrence, and mortality [4, 5]. Despite the potential improvement in survival outcomes, evidence suggests that adherence is low [6–12]. Multiple studies point to AET-related adverse symptoms as a key reason for non-adherence or premature discontinuation [7, 8, 10, 13–20]. In addition to adverse symptoms, other potentially modifiable factors that impact AET adherence include poorer patient-provider communication, fewer perceived treatment benefits, and barriers such as high cost and inconvenience [21].

Patients who do not take the full amount of their AET medication as prescribed or who discontinue their AET treatment early do not receive the full intended treatment benefits and, consequently, are at increased risk for mortality [22–25]. Observational studies have found significantly lower adherence [7, 26, 27] to AET among Black women when compared to White women. Racial differences in AET adherence may contribute to mortality disparities between Black and White women with breast cancer [6]. This challenge is magnified in Memphis, Tennessee, which has one of the highest mortality disparities in breast cancer survival in the country [28]. Survival differences by race persist even after controlling for stage at diagnosis, insurance status, income, and comorbidities [28–30]. It is possible that an intervention that standardizes patient-provider communication regarding adherence and symptom management may be able to reduce racial disparities in AET adherence and improve patient health outcomes.

For women with breast cancer in the adjuvant phase, clinic visits become less frequent. Because of the infrequency of clinic visits and limitations of patient recall, adverse symptoms are often not optimally evaluated or managed [13, 21]. Monitoring adverse symptoms, especially between clinic visits, could help healthcare providers better manage AET treatments of breast cancer patients without relying on patient recall. A few studies investigated the feasibility of symptom monitoring among oncology patients using web portals and automated telephone calls and found promising results [31–33]. However, these studies focused on alleviating symptoms, not improving medication adherence. Despite the critically important role of AET in reducing recurrence and mortality among women with HR+ breast cancer [2], only seven behavioral interventions to date have been specifically aimed toward increasing AET adherence [34–41]. Of these interventions, most provided educational materials only [37, 38, 41, 42] and only one showed a statistically significant improvement in adherence [36]. While there is some early evidence to suggests that communication between doctors and patients is associated with higher AET adherence, none of the AET adherence interventions focused on improving communication outside of clinic visits. Furthermore, there is a critical need for evidence on the impact of these types of interventions with underserved patients [43].

In our pilot trial, which used a web-based, electronic health record (EHR)-integrated app intervention designed to support adherence among women initiating AET treatment, participants receiving weekly reminders to use the study app reported significantly higher AET adherence at eight weeks compared with controls (91% vs. 68%, *p* = 0.02) [36]. The ability of healthcare providers to monitor symptom reports via an app and engage in treatment-related communication with patients outside of clinic visits could provide a wide-reaching and potentially cost-effective way to improve symptom management and ultimately health outcomes.

Our study tests a web-enabled app designed to improve patient-provider communication about AET adherence and related adverse symptoms outside of clinic visits. This builds on the success of our pilot study by: 1) expanding the intervention period from two to six months in order to capture later-onset adverse symptoms that might be slower to develop; 2) following participants for one year, to test longer-term effects of the intervention on medication adherence and other outcomes; and 3) including a larger sample, stratified by race, powered to test the intervention with and without tailored feedback messages.

## Methods

THRIVE is a five-year study funded by the National Cancer Institute. During the first year, we completed five focus groups to refine the study protocol, app functions and content, and develop feedback messages [44]. In the second year, we launched the randomized controlled trial of a web-based app and tailored messages for women with breast cancer initiating AET. The intervention lasts 6 months, and the primary end point is medication adherence assessed 12-months after enrollment.

### Aims of the study

1. Test if the App and App+Feedback conditions improve AET adherence.
2. Test if the App and App+Feedback conditions improve symptom burden, quality of life, patient-provider communication, and self-efficacy for managing symptoms.
3. Calculate the relative impact of the App and App+Feedback conditions on healthcare utilization and cost.

#### Conceptual framework

**Fig. 1:**
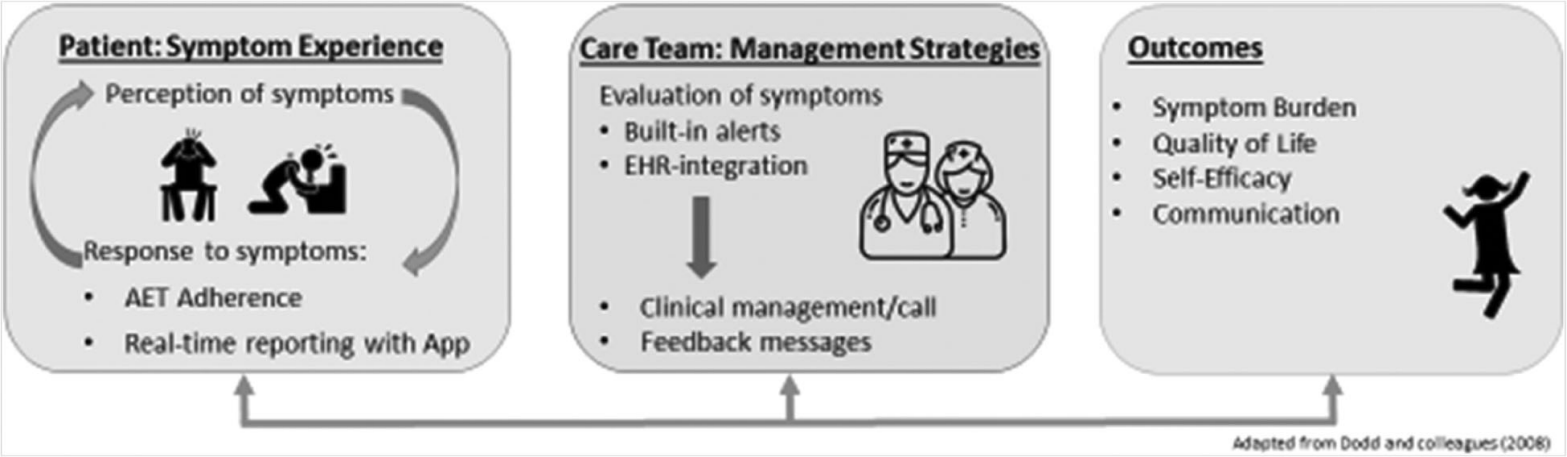
Conceptual Framework Guiding THRIVE Intervention

The intervention design was guided by the Symptom Management Model (Fig. 1), which describes the interrelatedness of three symptom management dimensions: symptom experience, management strategies, and health outcomes [45]. The model assumes that the patient’s perception of symptoms is the gold-standard of measurement, troublesome symptoms must be monitored and managed in a timely manner, and symptom management is dynamic [45]. An effective approach to improving treatment outcomes is one that is sensitive to an individual’s perception of her own symptoms, relies on ongoing communication and shared decision-making, and is patient-centered. Accordingly, we utilize real-time reporting of symptoms between patients and their oncology care teams outside of clinic visits. Built-in, real-time alerts and EHR-integration allows any patient’s report of AET nonadherence or adverse symptoms to be continually evaluated by the patient’s West Cancer Center Research Instiute (WCCRI) oncology team (i.e., the participant’s physician and their nursing team). In turn, providers have the opportunity to promptly address symptoms and other patient concerns, resulting in improved patient symptom experience and AET adherence. This cyclical process provides the patient and their clinical team the opportunity to communicate, evaluate, and manage symptoms.

The feedback component is based on self-regulation theory and will enhance patient engagement and activation and facilitate shared decision-making [46–48]. Cornerstones of any behavioral program are objective feedback and positive reinforcement [49–51]. The feedback serves to reinforce positive behavior in adherence and self-monitoring using the app. Evidence on app-based behavioral interventions supports the importance of tailored feedback to maintain self-monitoring behavior and optimize outcomes [49–53].

#### Study setting

Study participants will be enrolled at the WCCRI in Memphis, Tennessee. The WCCRI is the largest comprehensive oncology center in the tristate area of West Tennessee, Northern Mississippi, and East Arkansas, with a network of 14 clinic locations providing fully integrated cancer care. The WCCRI treats more than 1200 patients with a new breast cancer diagnosis each year. The WCCRI serves a diverse patient population similar to the surrounding region: nearly 40% of patients identify as racial/ethnic minorities, the majority of whom identify as Black.

#### Participants

Potentially eligible patients from all WCCRI locations are identified by our research nurse using WCCRI’s EHR system and by physician referral. Patients referred to the study meet with a research nurse who confirms eligibility, obtains informed consent, provides the electronic pillbox device, and has participants complete a baseline survey. Between November 2018 and March 2021, 300 participants will be recruited; participants complete study tasks for a minimum of a year (the primary study end-point), and up to 36 months, depending on how early in the trial they were enrolled. This study received approval from the University of Tennessee Health Science Center Institutional Review Board (IRB #: 17–05479-XP IAA).

#### Inclusion/exclusion criteria

The criteria for entry into the study include: a) adult female WCCRI patients (ages 18 years and older) with a diagnosis of ductal carcinoma in situ or Stage I-III hormone receptor-positive breast cancer; b) new prescription filled within the previous 8 weeks for an aromatase inhibitor (AI) or tamoxifen; c) have a mobile device with a data plan; d) have a valid email address; e) willing to complete brief surveys on a web-enabled device. Given that side effects associated with AET are typically more severe when treatment is first initiated, we exclude patients who have prior AET use. Because of potential exacerbations of possible side-effects, we also exclude patients with a current diagnosis of rheumatoid arthritis or fibromyalgia. We also exclude patients with chronic narcotic usage. Further, we do not include participants concurrently undergoing surgery or chemotherapy so that we can best disentangle the source of the side effects caused by AET alone. Our survey and app are only available in English, thus we exclude participants who are unable to communicate in English. Aside from chemotherapy and surgery, participants are permitted to undergo radiation and receive other concomitant treatments.

### Procedures

Following informed consent, all participants complete a baseline survey using the REDCap (Research Electronic Data Capture) database assessing basic demographic information and baseline measures of key study outcomes (see Table 2). REDCap is a secure web-based application that has many useful features for creating and managing online research databases and surveys while ensuring data integrity, such as auditing trails and secure data import and export functions [54].

The study statistician (MK) generated the randomization sequence with SAS using race-stratified block randomization with equal allocation 1:1:1 with block size of six. Randomization will be implemented in the RedCap protocol database. Only the study statistician and the database manager have access to the randomization scheme.

The study coordinator (AP) will randomize participants into one of the three study arms: 1) App, 2) App+Feedback, or 3) Usual Care. Finally, the study research nurse orients participants to their assigned condition and provides new enrollees with the study materials for their condition. All participants are given an electronic pill monitor (i.e., a WisePill device) and asked to use it for 12 months. Patients are asked to use this device exclusively with their prescribed AET medication. At enrollment, participants are trained on the use of the pillbox device, including refill instructions. If participants do not use the WisePill device for 14 consecutive days, study staff contact the participant via text or e-mail to troubleshoot barriers to device use.

**Fig. 2:**
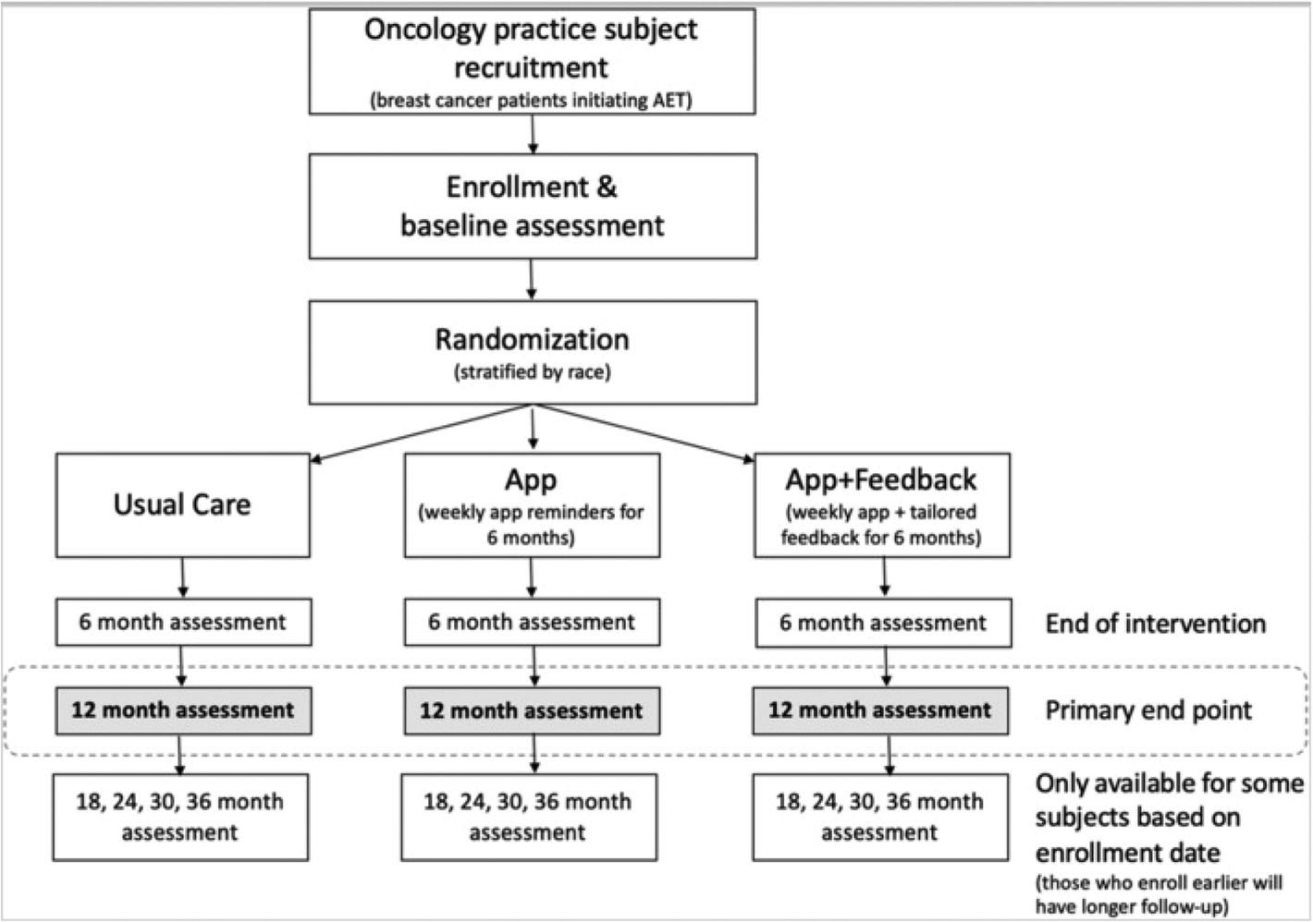
Patient flow through the RCT

At the 12-month visit, participants are asked to return their pillbox monitors either in person or using a preaddressed envelope, which is mailed to them. After returning their pill monitors at the end of month 12, participants are given a financial incentive to compensate them for the time and effort required to use the WisePill monitor. Figure 2 is a flow diagram of patient enrollment, randomization, and assessments through the trial.

Additionally, participants are emailed a secure link and asked to complete brief follow-up surveys every six months for the duration of the trial.

The participants randomized to the Usual Care arms are asked to use and return the WisePill box, and complete the surveys like the other two arms, but otherwise do not have any additional contact with the study team.

### App condition

**Fig. 3:**
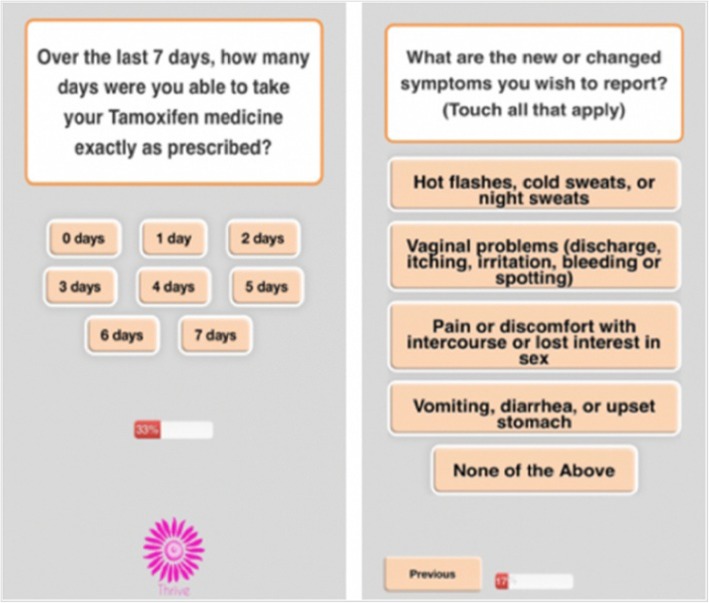
Screenshot of App Adherence and Symptom Questions

**Fig. 4:**
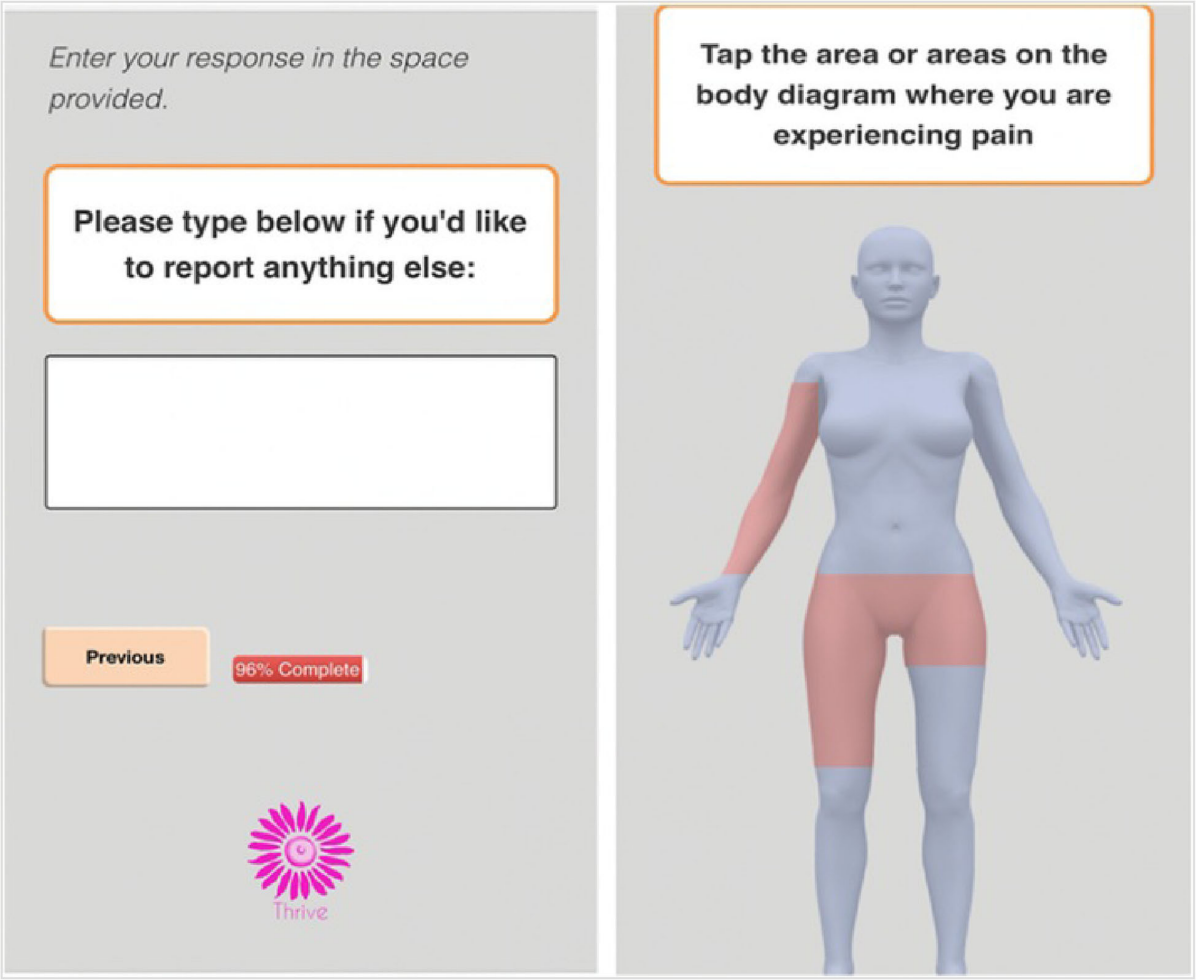
Screenshot of Free Text and Body Map

Participants in the App group receive a weekly text message to prompt them to log into the THRIVE app to answer questions about their AET adherence and related adverse symptoms. The app can be accessed through any web-enabled device or Internet browser. The reporting of medication use is assessed by a single-item adherence measure adapted from the Medication Adherence Reasons Scale [55, 56]. Symptoms are assessed using a condensed version of the FACT-ES [57], with follow-up questions about severity of symptoms using a 10-point severity scale. Figure 3 shows select screenshots of the THRIVE app’s symptom burden and adherence items. If participants select “Pain” as a symptom, they are prompted with a body map where they can indicate the specific area of the body in which they are experiencing pain (see Fig. 4). The questions are designed to limit the time burden on patients; reports should take about one to two minutes to complete.

During the formative phase of the study, we conducted focus groups with Black and White women with breast cancer to determine the optimal frequency (e.g., weekly, every other week, or monthly) and timing of messages and content (e.g., motivations, app use, AET adherence, symptom summary, relevant educational materials) [44]. These focus groups served to inform our intervention design, primarily through presentation of content to participants and allowing them to rate and provide feedback on both quality of content, utility of certain features, design aesthetic, and other methodological considerations. Examples of some of these findings include preferences for once-a-week reminder messages; a loss aversion scheme for incentivization; and the free text, body map, and patient dashboard features. Although participants are prompted to use the app at least once per week, they are informed that they may use it at any time regardless of when they receive prompts. All patient-reported data are automatically entered into the EHR system and easily available to oncology care teams for review. Furthermore, participants can use the free-text feature to convey any information they want to share with their provider that is not captured in the questions asked in the app (see Fig. 4).

The app alerts are based on response thresholds to adherence and symptom questions and are generated to inform the participant’s care team (i.e., prescribing physician and nurse) of any concerning responses or trends that emerge from the participant-reported outcomes via the app. WCCRI oncologists guided the alert thresholds, which include: three missed doses within the last week, a 4-point increase in symptom burden on the severity scale, or a score of 7–10 on the severity scale. Participants are also able to provide a free-text response to report anything they would like at the end of the survey, which is reviewed by the nurse coordinator who determines if a response from the oncology team is required.

These alerts inform providers of potentially concerning symptoms that warrant care team contact with the patient. The alert messages include the event that triggered the alert and are sent to the care team via e-mail. Care teams are asked to respond to alerts within 48 h. They are able to review concerning responses directly from the patient’s EHR to help guide ongoing treatment and make therapeutic adjustments when necessary. AET nonadherence for three days or more within one week prompts a call, even when the cause is not related to an adverse symptom. This facilitates communication between patients and their team on barriers to AET adherence and provides an opportunity for shared decision-making. A research nurse notes the clinical response and care team contact in the patient’s record after each alert (i.e., a phone consult, visit, and any medication changes).

### App+feedback condition

**Fig. 5:**
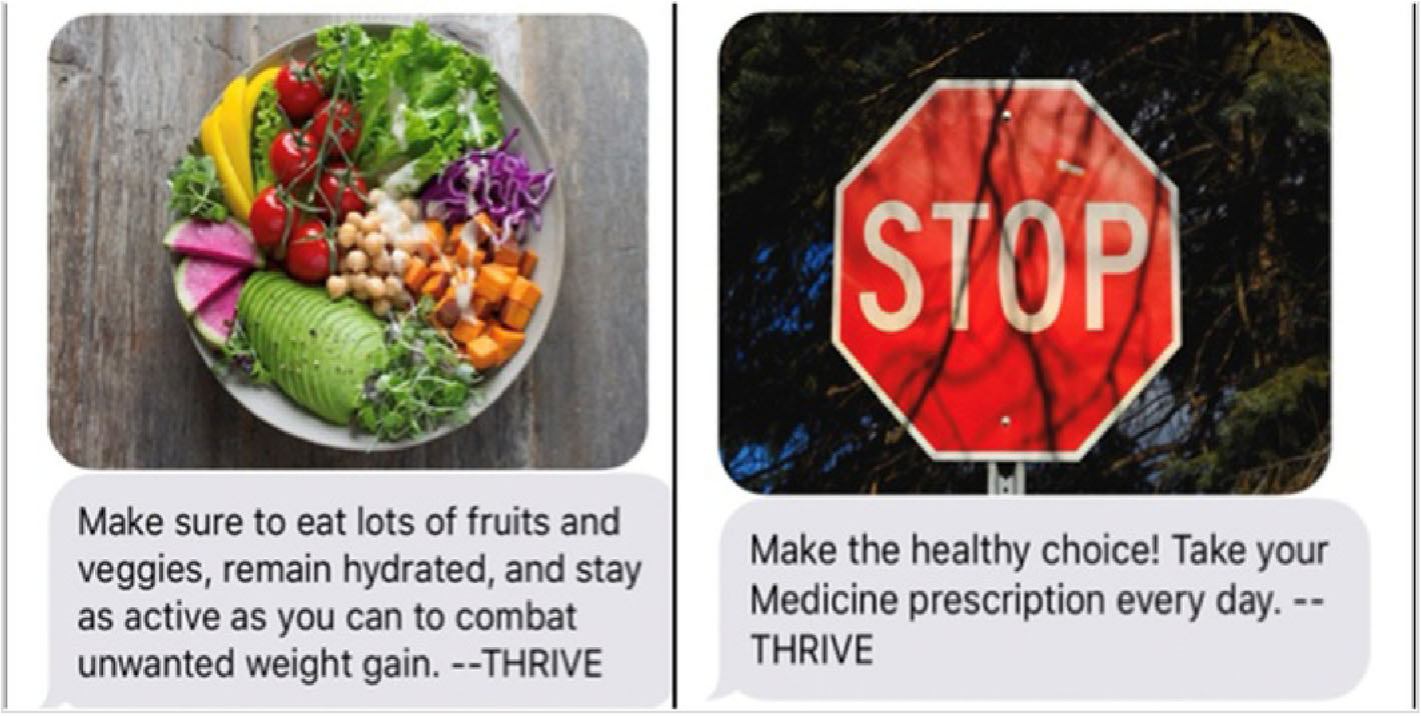
Screenshots of tailored app feedback messages

In addition to the previously outlined procedures, participants randomized to the App+Feedback group will receive weekly tailored feedback messages based on their baseline survey responses and use of the app during the 6-month intervention phase. Some tailored feedback includes links to symptom-specific educational materials and coping strategies for participants who report low-severity symptoms. Using feedback from focus groups, we developed a library of messages with multiple options for each condition in order to prevent desensitization to the same message. Message categories are tailored to participant’s responses to the app and baseline survey. Some feedback messages are supplemented with images (see Fig. 5).

### Data collection

#### Medication adherence monitoring

The WisePill monitor consists of a pillbox that wirelessly transmits adherence data each time the device is opened. The device uses mobile phone and Internet technologies to provide real-time adherence data. WisePill has been used to track medication adherence for other medications used to treat various diseases [58, 59].

#### Surveys

Participants are asked to complete brief online surveys every 6 months during their participation in the trial. Participants who do not complete the online follow-up surveys are offered other modalities to improve response rates, including mailing a paper version with a self-addressed return envelope or via phone interview with the research nurse. We will collect the following survey measures as covariates or secondary outcomes: a) a brief three-question, validated instrument to assess health literacy [60]; b) the Health Beliefs and Medication Adherence in Breast Cancer (HBMABC) [21] questionnaire, which focuses on patients’ perceived susceptibility to breast cancer recurrence and perceived benefits and barriers of AET; c) Self-Efficacy for Managing Symptoms- Short Form instrument [61]; d) Communication: Patient and Physician Peer Assessment Module 10-items instrument [62]; e) Wheeler et al.’s shared decision-making questions [63]; f) sociodemographic characteristics, including education level, relationship status, religious identity, household size, and household income; g) self-reported medication adherence, measured via a modified version of the Medication Adherence Questionnaire (MAQ) [64] and the Medication Adherence Rating Scale 1-item (MARS-1) [65]; and h) cost, measured via self-reported healthcare utilization (i.e., number of clinic visits, urgent care or emergency visits, hospitalizations, etc). Table 1 describes the key surveys and the relevant time points.

**Table 1:** Key Survey Measures and Data Collection Timeline

		Survey Time Points		
Measure	Description of Measure	Baseline	12-month Follow-Up	18+ Month Follow-up
Demographics	Age, highest level of education, total household income, race/ethnicity, current relationship status, sexual orientation, gender identity, and religious identity will be collected at baseline	X		
AET medication Adherence (adapted from MAQ) and MARS-1	Self-reported medication adherence will be measured via a modified version of the Medication Adherence Questionnaire (MAQ) [64] and the Medication Adherence Rating Scale 1-item (MARS-1) [65]		X	X
Symptom burden (FACT-ES)	The Functional Assessment of Cancer Therapy Endocrine Symptoms (FACT-ES), a 18-item instrument evaluates endocrine symptoms on a five-point Likert scale [57]	X	X	X
Quality of life (SF-12)	The Short-Form Health Survey (SF-12), a 12-item instrument that provides summary measures of physical and mental health status will be used [66, 67]	X	X	X
Communication: Patient and Physician Peer Assessment Module; Adapted 1-item measure	The Patient and Physician Peer Assessment Module [62] is an 11-item instrument that assesses patient perceptions of communication with their provider on scale of 1 (poor) to 5 (excellent). Shared decision making will also be assessed using a 1-item question adopted from Wheeler et al. [63]	X	X	X
Self-efficacy for managing symptoms (PROMIS)	The 4-item PROMIS Item Bank v1.0 – Self-Efficacy for Managing Symptoms short form scale will be used to measure confidence in a participant’s ability to successfully perform specific tasks or behaviors related to her health in a variety of situations [61]	X	X	X
Healthcare utilization in previous 6-months	A measure developed for this study based on the National Health Institutes Survey (NHIS) [68] will be used to assess healthcare utilization throughout the duration of the study.	X	X	X

#### Electronic health record chart abstraction

The following baseline demographic data are abstracted from the patient’s electronic health record upon enrollment:

- Date of birth, race/ethnicity, marital status, comorbidities
- Disease stage, tumor histology and grade, hormone receptor and human epidermal growth factor-2 (HER-2) status, menopausal status
- AI therapy and modalities of breast cancer treatment received in the primary adjuvant setting
- Chemotherapy and/or surgery received prior to AET
- Provider responses to the alerts

### Intervention fidelity monitoring and data management

We adhere to the following quality procedures to ensure treatment fidelity: 1) development of detailed intervention standard operating procedures; 2) electronic monitoring of receipt of emails/texts, app usage, and feedback reports; 3) documentation of all intervention contacts; and 4) weekly meetings to review overall adherence to structured protocols, and problem solving for any issues related to participant challenges. The study coordinator performs weekly data quality checks, and the study statistician performs range checks for data values on a monthly basis. All identifiable data is stored on password protected servers or locked file cabinets that only the study team has access to. The study PI, the study statistician, and the database manager will have access to the data. Quarterly reports will be generated from the accumulating data and the study team will carefully review the data for missingness and type of missingness and accuracy in data capture, and will develop timely action plans when necessary.

If any safety adverse events are discovered, a safety protocol will be followed according to standardized procedures used by the WCCRI as standard of care under the guidance of Drs. Schwartzberg and Vidal. All unanticipated adverse events will be recorded in a form that includes event date, whether the event is treatment related, and date event was addressed. The form will be given to Dr. Graetz within 24 h of learning of the event and the event documented by the appropriate staff member in progress notes, and reported to IRB, if appropriate. Alerts, adverse events, and referrals will not cause a participant to be dropped from the study, but will be considered in analysis. Any contact outside planned study contact will be documented (time, reason, actions taken, initiator) on the Delivery Assessment tracking form.

Any changes to the protocol are communicated to the proximal study team on a weekly basis, and to the wider study team on a monthly basis.

#### Study retention

The pilot study retention rate at eight weeks was 88% without the use of financial incentives to compensate patients for their time and contribution to the study. This high rate may reflect the proactive approach that we have employed, including maintaining current contact information, minimizing barriers by offering multiple modalities to complete surveys, and regular study meetings with retention as a standing agenda item. For this study, we are following participants for a longer period, which could impact retention rates; to maintain high retention rates, we provide small but meaningful incentives through a loss aversion scheme, as recommended by participants during the formative research phase. This includes a total compensation up to $220 in merchant credit depending on when they first enroll and how many follow-up surveys they complete. Specifically, for months 0–6, all participants are credited $60 merchant credit at the end of the period if they complete all activities, including the 6-month survey and maintenance of the WisePill device’s battery charge. For months 7–12, participants receive an additional $120 merchant credit for the second follow-up survey, WisePill battery charge maintenance, and return of the WisePill device. For months 18–36, participants who enroll early are asked to complete follow-up surveys every 6 months and receive $10 per survey, for a maximum of $40. Table 2 summarizes the compensation scheme for the study.

**Table 2:** THRIVE RCT Compensation Scheme

Months 0–6	Months 7–12	Month 18	Months 24	Months 32	Months 36
Baseline Survey, WisePill charge maintenance	Follow-up survey, WisePill charge maintenance and Return	Follow-up Survey	Follow-up Survey	Follow-up Survey	Follow-up Survey
$60	$120	$10	$10	$10	$10
$1 deduction for every day survey is not completed after 14-day grace period					
$1 deduction for every 5-day period of electronic pillbox with no charge	$1 deduction for every 5-day period with no charge, $30 bonus if returned within 2 weeks				

#### Criteria for modifying allocated interventions

If a participant is discontinued from adjuvant therapy, she is asked to still complete the follow-up surveys, but no longer receives reminder or feedback messages nor completes the app surveys if she was allocated to the app or app+feedback conditions.

### Statistical analysis

#### Power/sample size

Using adherence results from our pilot study and assuming 60% adherence in the ‘Usual Care’ arm, 75% adherence in the ‘App’ arm, and 85% adherence in the ‘App+Feedback’ arm, 95% power to detect a significance adherence difference among the three arms will be achieved with a total of 240 evaluable participants (80 evaluable patients in each arm) with 5% Type-1 error rate. We increase the sample size to 100 for each arm for a total accrual of 300 participants to account for potential 1-year attrition up to 20%. The same sample size would also provide 90% power with 5% Type-1 error rate to significantly detect the Quality of Life difference of 9.3 units where the ‘App’ and ‘App+Feedback’ arms are combined against the ‘Usual Care’ arm with projected standard deviations from the pilot study, of 9.3 and 24.3, respectively.

### Primary outcome

#### Adherence

Using the electronic pill monitoring system data, adherence will be defined as the proportion of days in which each participant took her medication (as recorded and transmitted via WisePill device opening) according to the prescribed frequency during the 12-month study period. For example, a patient would be considered to be 100% adherent if the pill monitoring electronic data showed that the box was opened on 365 days. Days during which patients were hospitalized will be deducted from the denominator. Persistence is commonly defined as the duration from AET initiation to discontinuation of the medication. Persistence will be calculated as the number of days from initiation until the first day of a gap that is 30 days or longer. The electronic medication monitoring system will be used solely as an outcome measure to compare AET adherence among the study groups, and it will not be used in the app, clinic alerts, or feedback reports.

### Secondary outcomes and covariates

### Quality of life, patient-provider communication, self-efficacy for managing symptoms, and symptom burden

Descriptive statistics of baseline characteristics on all participants will be presented and compared among the three treatment groups as means and standard deviations for continuous variables, and as frequencies and percentages for categorical variables, for each stratum, namely, White and Black strata, and for the combined data across strata. One-way analysis of variance (ANOVA) and t-tests for pairwise comparison, or its non-parametric counterparts such as Kruskal-Walls (or Wilcoxon-Mann-Whitney) test when warranted, and chi-squared or Fisher’s exact tests will be used to compare the groups for any differences in characteristics. We will also construct models utilizing the across-strata data to formally test for treatment and race interaction. The primary data analysis will adhere to the intent-to-treat principle.

#### Healthcare utilization and costs

To ensure completeness, we will combine self-reported healthcare utilization (reported every 6 months) with data abstracted from patient EHRs. Duplicates will be eliminated, and if conflicting information arises, we will assume that the EHR is correct. All utilization will be converted to cost using Medicare reimbursement rates. Using Medicare payment rates is a relatively standard approach in economic analysis because these rates provide a common metric for costing out services across all sectors of care (public or private) [48]; this is important because we are actually interested in the underlying resource utilization, not differences in prices. Medicare is also a major payer in all health care markets; almost all payers ‘follow Medicare’s lead’ when determining payment rates. Finally, many Medicare reimbursement rates were originally determined based on cost studies; thus, the Medicare relative fee structure bears some resemblance to the underlying relative cost structure.

For analysis of healthcare utilization data, we will employ two-part Hurdle Poisson models that are appropriate for “rate” data (e.g., visits per year). If we find that the data are over-dispersed, we will also explore negative binomial models. These models will yield two sets of coefficients, one related to the probability of any utilization and the other related to the level of utilization conditional on having any utilization. For cost data, we will employ two-part lognormal models to accommodate the high level of skewness. Again, this approach will yield two sets of coefficients: one related to probability of any cost and the other related to level of cost (conditional on having any cost). We have successfully used this approach in previous studies. We will also use survey reports from providers at clinic staff to accurately estimate costs to implement and maintain each intervention. For relative cost-effectiveness, we will calculate 6- and 12-month incremental cost-effectiveness ratios, comparing App or App+Feedback versus Usual Care at 6 and 12 months.

#### Missing data

Missing data, especially relating to the primary objective of the study, will be evaluated while the study is ongoing to develop corrective actions if possible. Once the study is completed, the missing data structure will be assessed and appropriate imputation approaches will be implemented if necessary.

### Dissemination plans

All objectives of the study, primary and secondary, will be addressed using the final study data and the results will be disseminated via article publications, conference presentations, and local seminars. In addition, a summary of the results will be shared on ClinicalTrials.gov. We do not expect that there will be an open access to the study data from the public.

## Discussion

The THRIVE Project is a three-arm (Usual Care, App Only, App+Feedback) randomized controlled trial, which examines the impact of an EHR-integrated, web-based communication app on women with early-stage breast cancer who initiate AET. We use messages and tailored feedback which are responsive to participant-reported symptom burden, and staff direct alerts to providers about concerning symptom and nonadherence. Given past research demonstrating that negative side effects are the leading cause of AET nonadherence [11], leveraging technologies such as the THRIVE app that connect patients with their care teams to facilitate timely changes to clinical management may reduce symptom burden and promote adherence. Other ongoing trials are similarly testing behavioral interventions to increase AET adherence [69, 70], using interactive messaging and nurse-delivered self-management training telephone call to improve symptom coping and side-effect management. An examination of the relative differences between results will be of great interest. Our results could advance the understanding of how real-time longitudinal capture of patient-reported symptoms with and without tailored feedback messages may aid patients and providers over the course of AET treatment.

There are several implications that could result from findings in this trial. If our hypotheses are correct, there are several potential contributions that would provide utility in clinical and research contexts. First, this study’s results may provide meaningful, real-time adherence data that will be used to assess factors associated with better or worse adherence and which kinds of interventions (e.g., communication app, or app plus tailored messages) are optimal for fostering higher adherence. Second, if successful, this intervention could be adapted for populations with other chronic conditions, including those in which medication adherence and self-management behaviors are crucial to preventing negative outcomes (e.g., diabetes, hypertension, human immunodeficiency virus [HIV]). Third, by evaluating the impact of this intervention on a comprehensive set of measures, including adherence, patient outcomes, racial disparities, and resource use-related costs, our study may provide valuable and actionable results for providers, policy makers, and insurers who strive to achieve the “Triple Aim”: reducing costs while improving health outcomes and the patient experience. Finally, a successful web-enabled intervention could be disseminated across healthcare systems to address AET adherence for women outside of the WCCRI.

## Data Availability

NA

## Abbreviations

AET: Adjuvant endocrine therapy;
AI: Aromatase inhibitor;
EHR: Electronic health record;
WCCRI: West Cancer Center & Research Institute
